# 1-[5-(2*H*-1,3-Benzodioxol-5-yl)-3-(4-methyl­phen­yl)-2-pyrazolin-1-yl]ethanone

**DOI:** 10.1107/S1600536813008817

**Published:** 2013-04-13

**Authors:** Wan-Sin Loh, Ibrahim Abdul Razak, M. Abdul Rahiman, G. N. Ravikumar

**Affiliations:** a X-ray Crystallography Unit, School of Physics, Universiti Sains Malaysia, 11800 USM, Penang, Malaysia; b Department of PG studies in Chemistry, Government Science College, Hassan 573 201, India

## Abstract

In the title compound, C_19_H_18_N_2_O_3_, the pyrazoline ring is close to being planar (r.m.s. deviation = 0.035 Å) and subtends dihedral angles of 2.11 (8) and 82.63 (8)° with the *p*-tolyl and benzene rings, respectively. In the crystal, C—H⋯O and C—H⋯N hydrogen bonds link the mol­ecules, forming a three-dimensional network. A weak C—H⋯π inter­action involving the benzene ring is also observed.

## Related literature

For background to pyrazoline derivatives, see: Mamolo *et al.* (2003 ▶); Bansal *et al.* (2001 ▶); Manna *et al.* (2005 ▶); Ahn *et al.* (2004 ▶); Rajendra Prasad *et al.* (2005 ▶). For a related structure, see: Du (2009 ▶). For the stability of the temperature controller used for the data collection, see: Cosier & Glazer (1986 ▶).
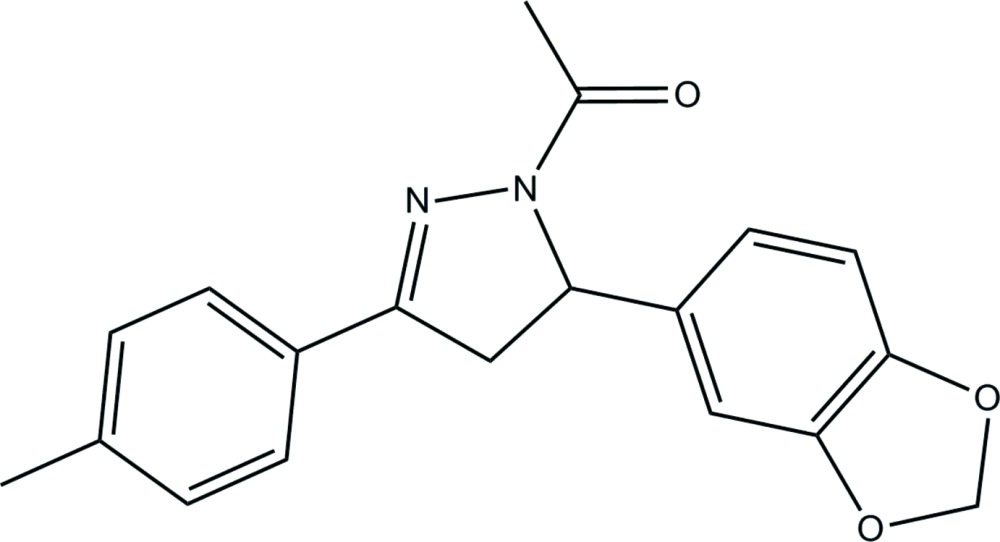


## Experimental

### 

#### Crystal data

C_19_H_18_N_2_O_3_
*M* *_r_* = 322.35
Monoclinic, 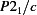

*a* = 7.9564 (1) Å
*b* = 24.1170 (4) Å
*c* = 8.4660 (1) Å
β = 105.380 (1)°
*V* = 1566.32 (4) Å^3^
*Z* = 4
Mo *K*α radiation
μ = 0.09 mm^−1^
*T* = 100 K
0.35 × 0.26 × 0.18 mm

#### Data collection

Bruker APEXII CCD diffractometer
Absorption correction: multi-scan (*SADABS*; Bruker, 2009 ▶) *T*_min_ = 0.968, *T*_max_ = 0.984
18850 measured reflections
4828 independent reflections
3778 reflections with *I* > 2σ(*I*)
*R*_int_ = 0.032

#### Refinement

*R*[*F* ^2^ > 2σ(*F* ^2^)] = 0.062
*wR*(*F* ^2^) = 0.136
*S* = 1.09
4828 reflections
219 parameters
H-atom parameters constrained
Δρ_max_ = 0.39 e Å^−3^
Δρ_min_ = −0.29 e Å^−3^

### 

Data collection: *APEX2* (Bruker, 2009 ▶); cell refinement: *SAINT* (Bruker, 2009 ▶); data reduction: *SAINT*; program(s) used to solve structure: *SHELXTL* (Sheldrick, 2008 ▶); program(s) used to refine structure: *SHELXTL*; molecular graphics: *SHELXTL*; software used to prepare material for publication: *SHELXTL* and *PLATON* (Spek, 2009 ▶).

## Supplementary Material

Crystal structure: contains datablock(s) global, I. DOI: 10.1107/S1600536813008817/hb7056sup1.cif

Structure factors: contains datablock(s) I. DOI: 10.1107/S1600536813008817/hb7056Isup2.hkl

Supplementary material file. DOI: 10.1107/S1600536813008817/hb7056Isup3.cml

Additional supplementary materials:  crystallographic information; 3D view; checkCIF report

## Figures and Tables

**Table 1:** Hydrogen-bond geometry (Å, °) *Cg*1 is the centroid of the C1–C3/C5–C7 benzene ring.

*D*—H⋯*A*	*D*—H	H⋯*A*	*D*⋯*A*	*D*—H⋯*A*
C2—H2*A*⋯O3^i^	0.95	2.42	3.229 (2)	142
C4—H4*A*⋯O3^ii^	0.99	2.38	3.225 (2)	143
C9—H9*B*⋯O2^iii^	0.99	2.59	3.371 (2)	136
C18—H18*B*⋯N2^iv^	0.98	2.56	3.526 (2)	169
C19—H19*A*⋯O3^v^	0.98	2.57	3.377 (2)	139
C19—H19*A*⋯*Cg*1^vi^	0.98	2.93	3.6013 (18)	127

Symmetry codes: (i) 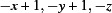; (ii) 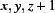; (iii) 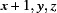; (iv) 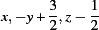; (v) 
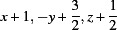
; (vi) 
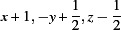
.

**Fig. 1.**
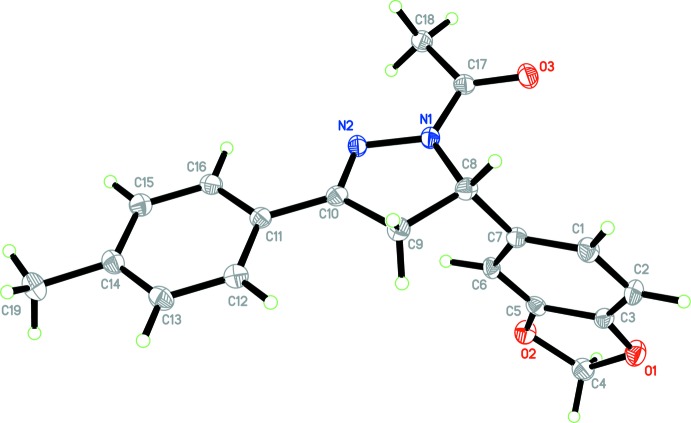
The molecular structure of the title compound, showing 50% probability displacement ellipsoids.

**Fig. 2.**
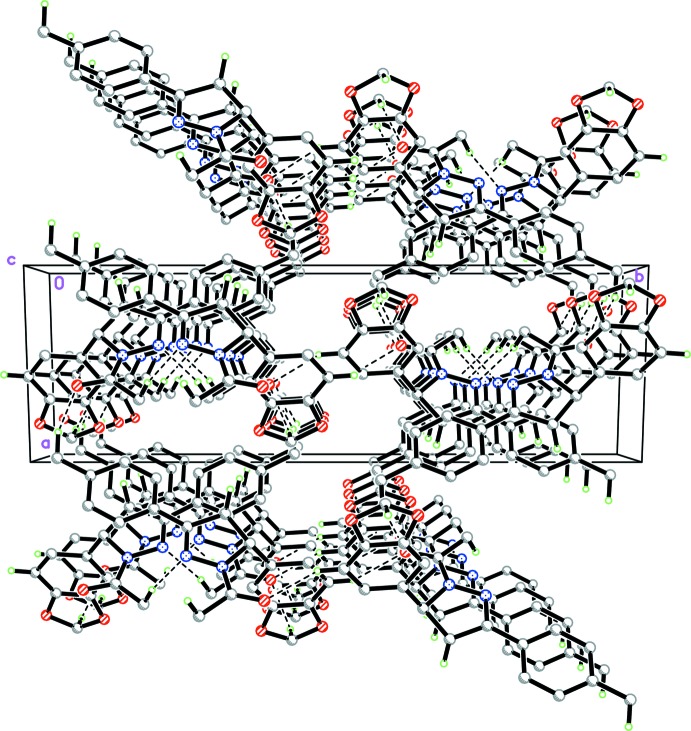
The crystal packing of the title compound, viewed along the c axis. H atoms not involved in the intermolecular interactions (dashed lines) have been omitted for clarity.

## supplementary crystallographic information

### Comment

Considerable attention has been focused on pyrazoline derivatives due to their interesting biological activities. Some of the pyrazoline derivatives are reported to possess antifungal (Mamolo *et al.*, 2003), anti-inflammatory (Bansal *et al.*, 2001), anticancer (Manna *et al.*, 2005), antidiabetic (Ahn *et al.*, 2004) and antidepressant (Rajendra Prasad *et al.*, 2005) properties.

In the title compound, Fig. 1, the benzodioxole ring system (C1–C7/O1/O2) and the pyrazole ring (C8–C10/N1/N2) are almost planar with maximum deviations of 0.081 (2) Å at atom C4 and 0.029 (2) Å at atom C8, respectively. They are almost perpendicular to each other with the dihedral angle of 82.08 (7)°. The pyrazole ring also forms dihedral angle of 2.11 (8)° with the benzene ring (C11–C16). Bond lengths and angles are almost comparable with the related structure (Du, 2009).

In the crystal, Fig. 2, C—H···O and C—H···N hydrogen bonds (Table 1) link the molecules to form three dimensional network and also feature C—H···π interactions (Table 1) involving the benzene ring (*Cg*1; C1–C3/C5–C7).

### Experimental

1-*p*-Tolyl-3(3',4'-methylene dioxyphenyl)-2-propen-1-one (0.005 mol) and hydrazine hydrate (0.005 mol) in acetic acid (15 ml) was refluxed for 6 h. The solid on cooling was collected and washed well with cold water and alcohol. It was dried and recrystallized from ethanol-dioxan. Colourless blocks were obtained by slow evaporation of a DMF/ethanol solution (1:2 *v/v*).

### Refinement

All the H atoms were located geometrically and were refined using a riding model with *U*_iso_(H) = 1.2 or 1.5 *U*_eq_(C) [C–H = 0.95 to 1.00 Å]. A rotating group model was applied to the methyl groups. In the final refinement, three outliners were omitted, 0 2 0, -7 13 4 and 9 13 1.

### Crystal data

**Table:**

C_19_H_18_N_2_O_3_	*F*(000) = 680
*M**_r_* = 322.35	*D*_x_ = 1.367 Mg m^−^^3^
Monoclinic, *P*2_1_/*c*	Mo *K*α radiation, λ = 0.71073 Å
Hall symbol: -P 2ybc	Cell parameters from 6808 reflections
*a* = 7.9564 (1) Å	θ = 2.6–30.6°
*b* = 24.1170 (4) Å	µ = 0.09 mm^−^^1^
*c* = 8.4660 (1) Å	*T* = 100 K
β = 105.380 (1)°	Block, colourless
*V* = 1566.32 (4) Å^3^	0.35 × 0.26 × 0.18 mm
*Z* = 4	

### Data collection

**Table:**

Bruker APEXII CCD diffractometer	4828 independent reflections
Radiation source: fine-focus sealed tube	3778 reflections with *I* > 2σ(*I*)
Graphite monochromator	*R*_int_ = 0.032
φ and ω scans	θ_max_ = 30.7°, θ_min_ = 2.6°
Absorption correction: multi-scan (*SADABS*; Bruker, 2009)	*h* = −11→7
*T*_min_ = 0.968, *T*_max_ = 0.984	*k* = −28→34
18850 measured reflections	*l* = −12→12

### Refinement

**Table:**

Refinement on *F*^2^	Primary atom site location: structure-invariant direct methods
Least-squares matrix: full	Secondary atom site location: difference Fourier map
*R*[*F*^2^ > 2σ(*F*^2^)] = 0.062	Hydrogen site location: inferred from neighbouring sites
*wR*(*F*^2^) = 0.136	H-atom parameters constrained
*S* = 1.09	*w* = 1/[σ^2^(*F*_o_^2^) + (0.0389*P*)^2^ + 1.3146*P*] where *P* = (*F*_o_^2^ + 2*F*_c_^2^)/3
4828 reflections	(Δ/σ)_max_ = 0.001
219 parameters	Δρ_max_ = 0.39 e Å^−^^3^
0 restraints	Δρ_min_ = −0.29 e Å^−^^3^

### Special details

**Table:**

Experimental. The crystal was placed in the cold stream of an Oxford Cryosystems Cobra open-flow nitrogen cryostat (Cosier & Glazer, 1986) operating at 100.0 (1) K.
Geometry. All e.s.d.'s (except the e.s.d. in the dihedral angle between two l.s. planes) are estimated using the full covariance matrix. The cell e.s.d.'s are taken into account individually in the estimation of e.s.d.'s in distances, angles and torsion angles; correlations between e.s.d.'s in cell parameters are only used when they are defined by crystal symmetry. An approximate (isotropic) treatment of cell e.s.d.'s is used for estimating e.s.d.'s involving l.s. planes.
Refinement. Refinement of *F*^2^ against ALL reflections. The weighted *R*-factor *wR* and goodness of fit *S* are based on *F*^2^, conventional *R*-factors *R* are based on *F*, with *F* set to zero for negative *F*^2^. The threshold expression of *F*^2^ > σ(*F*^2^) is used only for calculating *R*-factors(gt) *etc*. and is not relevant to the choice of reflections for refinement. *R*-factors based on *F*^2^ are statistically about twice as large as those based on *F*, and *R*- factors based on ALL data will be even larger.

### Fractional atomic coordinates and isotropic or equivalent isotropic displacement parameters (Å^2^)

**Table:**

	*x*	*y*	*z*	*U*_iso_*/*U*_eq_	
O1	0.20199 (17)	0.52198 (5)	0.40364 (15)	0.0242 (3)	
O2	0.18293 (16)	0.61783 (5)	0.38140 (14)	0.0204 (3)	
O3	0.40078 (16)	0.60685 (5)	−0.20460 (14)	0.0212 (3)	
N1	0.54641 (18)	0.67124 (5)	−0.03118 (16)	0.0164 (3)	
N2	0.59601 (18)	0.72596 (5)	0.00463 (16)	0.0154 (3)	
C1	0.5458 (2)	0.54240 (7)	0.1907 (2)	0.0197 (3)	
H1A	0.6263	0.5232	0.1455	0.024*	
C2	0.4417 (2)	0.51184 (7)	0.2695 (2)	0.0212 (3)	
H2A	0.4504	0.4726	0.2796	0.025*	
C3	0.3269 (2)	0.54167 (7)	0.33134 (18)	0.0172 (3)	
C4	0.1270 (2)	0.57023 (7)	0.4575 (2)	0.0223 (4)	
H4A	0.1669	0.5735	0.5783	0.027*	
H4B	−0.0018	0.5676	0.4250	0.027*	
C5	0.3156 (2)	0.59905 (6)	0.31861 (18)	0.0157 (3)	
C6	0.4180 (2)	0.62926 (6)	0.24358 (18)	0.0156 (3)	
H6A	0.4103	0.6685	0.2371	0.019*	
C7	0.5353 (2)	0.59965 (6)	0.17627 (18)	0.0163 (3)	
C8	0.6489 (2)	0.63082 (7)	0.08798 (19)	0.0172 (3)	
H8A	0.7085	0.6040	0.0306	0.021*	
C9	0.7850 (2)	0.66945 (7)	0.19965 (19)	0.0178 (3)	
H9A	0.7825	0.6660	0.3155	0.021*	
H9B	0.9041	0.6611	0.1910	0.021*	
C10	0.7271 (2)	0.72633 (7)	0.13314 (18)	0.0156 (3)	
C11	0.8090 (2)	0.77833 (7)	0.20273 (18)	0.0157 (3)	
C12	0.9503 (2)	0.77802 (7)	0.34314 (19)	0.0181 (3)	
H12A	0.9944	0.7438	0.3928	0.022*	
C13	1.0259 (2)	0.82764 (7)	0.40982 (19)	0.0197 (3)	
H13A	1.1210	0.8268	0.5053	0.024*	
C14	0.9654 (2)	0.87859 (7)	0.34000 (19)	0.0185 (3)	
C15	0.8254 (2)	0.87857 (7)	0.19888 (19)	0.0184 (3)	
H15A	0.7829	0.9128	0.1481	0.022*	
C16	0.7481 (2)	0.82948 (7)	0.13228 (18)	0.0174 (3)	
H16A	0.6523	0.8305	0.0373	0.021*	
C17	0.4283 (2)	0.65621 (7)	−0.17337 (18)	0.0163 (3)	
C18	0.3369 (2)	0.70214 (7)	−0.28355 (19)	0.0188 (3)	
H18A	0.2313	0.6875	−0.3600	0.028*	
H18B	0.4147	0.7172	−0.3453	0.028*	
H18C	0.3051	0.7316	−0.2171	0.028*	
C19	1.0460 (2)	0.93221 (7)	0.4153 (2)	0.0246 (4)	
H19A	1.1729	0.9302	0.4352	0.037*	
H19B	1.0166	0.9383	0.5193	0.037*	
H19C	1.0008	0.9630	0.3404	0.037*	

### Atomic displacement parameters (Å^2^)

**Table:**

	*U*^11^	*U*^22^	*U*^33^	*U*^12^	*U*^13^	*U*^23^
O1	0.0277 (7)	0.0191 (6)	0.0290 (6)	−0.0026 (5)	0.0129 (5)	0.0007 (5)
O2	0.0195 (6)	0.0193 (6)	0.0251 (6)	0.0014 (5)	0.0105 (5)	−0.0008 (5)
O3	0.0242 (6)	0.0163 (6)	0.0221 (5)	−0.0015 (5)	0.0045 (5)	−0.0032 (4)
N1	0.0187 (7)	0.0131 (6)	0.0164 (6)	−0.0010 (5)	0.0030 (5)	0.0003 (5)
N2	0.0162 (7)	0.0136 (6)	0.0172 (6)	−0.0019 (5)	0.0061 (5)	−0.0016 (5)
C1	0.0212 (8)	0.0160 (7)	0.0217 (7)	0.0044 (6)	0.0055 (6)	−0.0013 (6)
C2	0.0261 (9)	0.0128 (7)	0.0242 (7)	0.0008 (6)	0.0059 (7)	0.0023 (6)
C3	0.0175 (8)	0.0174 (7)	0.0162 (7)	−0.0035 (6)	0.0031 (6)	0.0011 (6)
C4	0.0223 (9)	0.0236 (8)	0.0221 (7)	−0.0027 (7)	0.0078 (7)	−0.0021 (6)
C5	0.0140 (7)	0.0167 (7)	0.0148 (6)	0.0017 (6)	0.0010 (6)	−0.0023 (5)
C6	0.0164 (8)	0.0116 (7)	0.0173 (6)	0.0016 (6)	0.0017 (6)	−0.0012 (5)
C7	0.0170 (8)	0.0157 (7)	0.0146 (6)	−0.0005 (6)	0.0016 (6)	0.0006 (5)
C8	0.0172 (8)	0.0163 (7)	0.0177 (7)	0.0025 (6)	0.0042 (6)	0.0012 (6)
C9	0.0151 (8)	0.0182 (7)	0.0193 (7)	−0.0003 (6)	0.0032 (6)	0.0011 (6)
C10	0.0152 (8)	0.0179 (7)	0.0155 (6)	−0.0006 (6)	0.0072 (6)	0.0000 (6)
C11	0.0139 (7)	0.0191 (7)	0.0156 (6)	−0.0018 (6)	0.0067 (6)	−0.0009 (6)
C12	0.0156 (8)	0.0199 (8)	0.0192 (7)	0.0012 (6)	0.0053 (6)	0.0003 (6)
C13	0.0154 (8)	0.0241 (8)	0.0185 (7)	−0.0011 (6)	0.0023 (6)	−0.0031 (6)
C14	0.0170 (8)	0.0200 (8)	0.0197 (7)	−0.0015 (6)	0.0068 (6)	−0.0041 (6)
C15	0.0190 (8)	0.0178 (8)	0.0190 (7)	0.0019 (6)	0.0060 (6)	−0.0005 (6)
C16	0.0163 (8)	0.0208 (8)	0.0152 (6)	−0.0011 (6)	0.0042 (6)	−0.0005 (6)
C17	0.0169 (8)	0.0175 (7)	0.0165 (6)	−0.0001 (6)	0.0080 (6)	−0.0017 (6)
C18	0.0188 (8)	0.0189 (8)	0.0177 (7)	−0.0010 (6)	0.0028 (6)	0.0003 (6)
C19	0.0242 (9)	0.0218 (8)	0.0263 (8)	−0.0016 (7)	0.0041 (7)	−0.0060 (7)

### Geometric parameters (Å, º)

**Table:**

O1—C3	1.382 (2)	C9—C10	1.508 (2)
O1—C4	1.436 (2)	C9—H9A	0.9900
O2—C5	1.378 (2)	C9—H9B	0.9900
O2—C4	1.443 (2)	C10—C11	1.463 (2)
O3—C17	1.2265 (19)	C11—C16	1.399 (2)
N1—C17	1.3651 (19)	C11—C12	1.403 (2)
N1—N2	1.3877 (18)	C12—C13	1.390 (2)
N1—C8	1.482 (2)	C12—H12A	0.9500
N2—C10	1.292 (2)	C13—C14	1.393 (2)
C1—C7	1.387 (2)	C13—H13A	0.9500
C1—C2	1.403 (2)	C14—C15	1.401 (2)
C1—H1A	0.9500	C14—C19	1.508 (2)
C2—C3	1.371 (2)	C15—C16	1.383 (2)
C2—H2A	0.9500	C15—H15A	0.9500
C3—C5	1.389 (2)	C16—H16A	0.9500
C4—H4A	0.9900	C17—C18	1.506 (2)
C4—H4B	0.9900	C18—H18A	0.9800
C5—C6	1.369 (2)	C18—H18B	0.9800
C6—C7	1.410 (2)	C18—H18C	0.9800
C6—H6A	0.9500	C19—H19A	0.9800
C7—C8	1.516 (2)	C19—H19B	0.9800
C8—C9	1.546 (2)	C19—H19C	0.9800
C8—H8A	1.0000		
			
C3—O1—C4	105.69 (13)	C10—C9—H9B	111.2
C5—O2—C4	105.61 (13)	C8—C9—H9B	111.2
C17—N1—N2	122.31 (13)	H9A—C9—H9B	109.1
C17—N1—C8	123.46 (13)	N2—C10—C11	121.24 (14)
N2—N1—C8	113.80 (12)	N2—C10—C9	113.99 (14)
C10—N2—N1	108.04 (13)	C11—C10—C9	124.76 (13)
C7—C1—C2	122.41 (16)	C16—C11—C12	118.35 (14)
C7—C1—H1A	118.8	C16—C11—C10	121.13 (14)
C2—C1—H1A	118.8	C12—C11—C10	120.52 (14)
C3—C2—C1	116.31 (15)	C13—C12—C11	120.18 (15)
C3—C2—H2A	121.8	C13—C12—H12A	119.9
C1—C2—H2A	121.8	C11—C12—H12A	119.9
C2—C3—O1	128.25 (15)	C12—C13—C14	121.57 (14)
C2—C3—C5	121.95 (16)	C12—C13—H13A	119.2
O1—C3—C5	109.69 (15)	C14—C13—H13A	119.2
O1—C4—O2	107.41 (13)	C13—C14—C15	117.95 (15)
O1—C4—H4A	110.2	C13—C14—C19	121.13 (14)
O2—C4—H4A	110.2	C15—C14—C19	120.92 (15)
O1—C4—H4B	110.2	C16—C15—C14	120.99 (15)
O2—C4—H4B	110.2	C16—C15—H15A	119.5
H4A—C4—H4B	108.5	C14—C15—H15A	119.5
C6—C5—O2	127.96 (14)	C15—C16—C11	120.96 (14)
C6—C5—C3	122.14 (15)	C15—C16—H16A	119.5
O2—C5—C3	109.77 (14)	C11—C16—H16A	119.5
C5—C6—C7	117.22 (14)	O3—C17—N1	119.32 (14)
C5—C6—H6A	121.4	O3—C17—C18	123.45 (14)
C7—C6—H6A	121.4	N1—C17—C18	117.23 (14)
C1—C7—C6	119.95 (15)	C17—C18—H18A	109.5
C1—C7—C8	120.50 (15)	C17—C18—H18B	109.5
C6—C7—C8	119.54 (14)	H18A—C18—H18B	109.5
N1—C8—C7	111.69 (13)	C17—C18—H18C	109.5
N1—C8—C9	100.92 (12)	H18A—C18—H18C	109.5
C7—C8—C9	114.17 (13)	H18B—C18—H18C	109.5
N1—C8—H8A	109.9	C14—C19—H19A	109.5
C7—C8—H8A	109.9	C14—C19—H19B	109.5
C9—C8—H8A	109.9	H19A—C19—H19B	109.5
C10—C9—C8	102.99 (12)	C14—C19—H19C	109.5
C10—C9—H9A	111.2	H19A—C19—H19C	109.5
C8—C9—H9A	111.2	H19B—C19—H19C	109.5
			
C17—N1—N2—C10	−170.21 (15)	C1—C7—C8—C9	−114.22 (16)
C8—N1—N2—C10	2.53 (18)	C6—C7—C8—C9	66.00 (18)
C7—C1—C2—C3	0.5 (2)	N1—C8—C9—C10	4.62 (16)
C1—C2—C3—O1	175.04 (15)	C7—C8—C9—C10	−115.32 (15)
C1—C2—C3—C5	−0.8 (2)	N1—N2—C10—C11	−179.57 (14)
C4—O1—C3—C2	175.73 (16)	N1—N2—C10—C9	1.01 (19)
C4—O1—C3—C5	−8.00 (17)	C8—C9—C10—N2	−3.82 (19)
C3—O1—C4—O2	13.04 (16)	C8—C9—C10—C11	176.78 (15)
C5—O2—C4—O1	−13.22 (16)	N2—C10—C11—C16	0.0 (2)
C4—O2—C5—C6	−175.69 (15)	C9—C10—C11—C16	179.32 (16)
C4—O2—C5—C3	8.42 (16)	N2—C10—C11—C12	179.46 (16)
C2—C3—C5—C6	0.1 (2)	C9—C10—C11—C12	−1.2 (2)
O1—C3—C5—C6	−176.47 (13)	C16—C11—C12—C13	0.4 (2)
C2—C3—C5—O2	176.25 (14)	C10—C11—C12—C13	−179.11 (15)
O1—C3—C5—O2	−0.30 (17)	C11—C12—C13—C14	−0.4 (3)
O2—C5—C6—C7	−174.44 (14)	C12—C13—C14—C15	−0.3 (3)
C3—C5—C6—C7	1.0 (2)	C12—C13—C14—C19	178.74 (16)
C2—C1—C7—C6	0.6 (2)	C13—C14—C15—C16	0.9 (3)
C2—C1—C7—C8	−179.21 (14)	C19—C14—C15—C16	−178.14 (16)
C5—C6—C7—C1	−1.3 (2)	C14—C15—C16—C11	−0.8 (3)
C5—C6—C7—C8	178.49 (13)	C12—C11—C16—C15	0.2 (2)
C17—N1—C8—C7	−70.29 (19)	C10—C11—C16—C15	179.70 (15)
N2—N1—C8—C7	117.07 (14)	N2—N1—C17—O3	173.77 (15)
C17—N1—C8—C9	168.00 (15)	C8—N1—C17—O3	1.7 (2)
N2—N1—C8—C9	−4.64 (17)	N2—N1—C17—C18	−6.8 (2)
C1—C7—C8—N1	132.07 (15)	C8—N1—C17—C18	−178.84 (15)
C6—C7—C8—N1	−47.70 (18)		

### Hydrogen-bond geometry (Å, º)

Cg1 is the centroid of the C1–C3/C5–C7 benzene ring.

**Table:**

*D*—H···*A*	*D*—H	H···*A*	*D*···*A*	*D*—H···*A*
C2—H2*A*···O3^i^	0.95	2.42	3.229 (2)	142
C4—H4*A*···O3^ii^	0.99	2.38	3.225 (2)	143
C9—H9*B*···O2^iii^	0.99	2.59	3.371 (2)	136
C18—H18*B*···N2^iv^	0.98	2.56	3.526 (2)	169
C19—H19*A*···O3^v^	0.98	2.57	3.377 (2)	139
C19—H19*A*···*Cg*1^vi^	0.98	2.93	3.6013 (18)	127

Symmetry codes: (i) −*x*+1, −*y*+1, −*z*; (ii) *x*, *y*, *z*+1; (iii) *x*+1, *y*, *z*; (iv) *x*, −*y*+3/2, *z*−1/2; (v) *x*+1, −*y*+3/2, *z*+1/2; (vi) *x*+1, −*y*+1/2, *z*−1/2.
